# *Salmonella* Vaccine Vector System for Foot-and-Mouth Disease Virus and Evaluation of Its Efficacy with Virus-Like Particles

**DOI:** 10.3390/vaccines9010022

**Published:** 2021-01-05

**Authors:** Yong Zhi, Hyun Jung Ji, Huichen Guo, Jae Hyang Lim, Eui-Baek Byun, Woo Sik Kim, Ho Seong Seo

**Affiliations:** 1 Research Division for Radiation Science, Korea Atomic Energy Research Institute, Jeongeup 56212, Korea; yongzhi@kaeri.re.kr (Y.Z.); hyunjung@kaeri.re.kr (H.J.J.); ebbyun80@kaeri.re.kr (E.-B.B.); 2 Department of Radiation Science, University of Science and Technology, Daejeon 34113, Korea; 3 Department of Oral Microbiology and Immunology, DRI, and BK21 Plus Program, School of Dentistry, Seoul National University, Seoul 08826, Korea; 4 State Key Laboratory of Veterinary Etiological Biology, National Foot and Mouth Disease Reference Laboratory, Lanzhou Veterinary Research Institute, Chinese Academy of Agricultural Sciences, Lanzhou 730000, China; guohuichen@caas.cn; 5 Department of Microbiology, Ewha Womans University College of Medicine, Seoul 07804, Korea; jlim19@ewha.ac.kr; 6 Ewha Education & Research Center for Infection, Ewha Womans University Medical Center, Seoul 07985, Korea; 7 Functional Biomaterial Research Center, Korea Research Institute of Bioscience and Biotechnology, Jeongeup 56212, Korea

**Keywords:** foot-and-mouth disease, VP1, live attenuated *Salmonella* vector, mucosal immunity, virus-like particle, radiation mutation

## Abstract

Foot-and-mouth disease virus (FMDV) causes a highly contagious and devastating disease in livestock animals and has a great potential to cause severe economic loss worldwide. The major antigen of FMDV capsid protein, VP1, contains the major B-cell epitope responsible for effectively eliciting protective humoral immunity. In this study, irradiated *Salmonella* Typhimurium (KST0666) were used as transgenic vectors containing stress-inducible plasmid pRECN-VP1 to deliver the VP1 protein from FMDV-type A/WH/CHA/09. Mice were orally inoculated with ATOMASal-L3 harboring pRECN-VP1, and FMDV virus-like particles, where (VLP_FMDV_)-specific humoral, mucosal, and cellular immune responses were evaluated. Mice vaccinated with attenuated *Salmonella* (KST0666) expressing VP1 (named KST0669) showed high levels of VLP-specific IgA in feces and IgG in serum, with high FMDV neutralization titer. Moreover, KST0669-vaccinated mice showed increased population of IFN-γ (type 1 T helper cells; Th1 cells)-, IL-5 (Th2 cells)-, and IL-17A (Th17 cells)-expressing CD4^+^ as well as activated CD8^+^ T cells (IFN-γ^+^CD8^+^ cells), detected by stimulating VLP_FMDV_. All data indicate that our *Salmonella* vector system successfully delivered FMDV VP1 to immune cells and that the humoral and cellular efficacy of the vaccine can be easily evaluated using VLP_FMDV_ in a Biosafety Level I (BSL1) laboratory.

## 1. Introduction

Foot-and-mouth disease (FMD) may result in serious economic losses to the livestock industry by causing abortions, weight loss, and reduced milk production [1,2]. FMD virus (FMDV) is a highly contagious pathogen that causes blisters inside the mouth and bullous lesions on the feet of cloven-hoofed animals [2]. FMDV is a positive-sense, single-stranded RNA (ssRNA) virus that belongs to the genus *Aphthovirus* and the family *Picornaviridae.* In total, seven serotypes (A, O, C, Asia 1, and South African Territories 1, 2, and 3) of the virus have been identified, and multiple subtypes occur within each serotype [2,3,4]. FMDV virion consists of an icosahedral capsid with twelve pentamers of the four structural polypeptides (VP1 to VP4) enclosing about 8.3 kb long ssRNA genome [5].

Vaccination has so far been the best strategy to prevent and suppress the FMD epidemic [6]. The current FMD vaccines that are commonly used in endemic areas contain inactivated whole-virus with binary ethyleneimine (BEI) or formaldehyde, followed by formulating with an oil-based adjuvant [7,8]. Although the currently available vaccines have been shown to reduce FMDV prevalence in endemic areas, they have several limitations: (1) the requirement of a Biosafety Level III (BSL3) facilities for mass production of the virus antigen, (2) extensive genetic variation during manufacturing process, (3) short-term immunity due to lower immunogenicity, (4) lower cross-protective immunity against heterogenous serotypes and subtypes [9,10]. In addition, other important concerns regarding inactivated vaccines include multiple vaccination, cold chains, and accidental viral release from manufacturing facility [11].

Various studies have been conducted to develop the next generation FMDV vaccines. Live attenuated vaccines were generated through natural mutations by adapting FMDV in suckling mice in the United Kingdom, but large-scale clinical trial failed due to incomplete attenuation [12,13]. As the capsid proteins of FMDV have potent immunogenic properties, the empty capsid virus-like-particles (VLPs) produced in the *Escherichia coli* (*E. coli*) or *Spodoptera frugiperda* (Sf21) insect cell system have been developed as safer alternative vaccine candidates [14,15,16]. In fact, FMDV VLPs (VLP_FMDV_) synthesized from *E. coli* reportedly generate similar levels of humoral and protective immune responses to those of current inactivated vaccines [17,18]. In general, recombinant conserved epitope of FMDV is another potential solution, owing to the availability of several highly cost-effective and safe protein expression systems [19,20]. As previous studies have confirmed the localization of multiple major antigenic sites in the G-H loop (amino acids 141–160) of the capsid protein VP1, VP1 or its short peptide has been extensively studied as a potent recombinant antigen [21,22,23]. Although these protein-based vaccines have several advantages, such as being economical and safe, their low cellular immune response has limited the commercialization of these vaccines.

An ideal vaccine to overcome the above limitations should be characterized by the ability to combine with immune modulation systems so as to activate pathogen-specific T cell immune responses [24,25]. Live replicating organisms are able to deliver immunogenic viral structural proteins by acting as natural adjuvants to stimulate the mucosal and cellular immune responses [21,26,27]. Human adenovirus type 5 vectors encoding the capsid protein precursor P1-2A of FMDV produced higher FMDV-specific IgG, CD4^+^, and CD8^+^ T cell responses than inactivated FMDV vaccine in immunized mice [28]. *Salmonella*, as an antigen transfer vector, is one of the most widely studied bacteria because it can invade the gut-associated lymphoid tissue and effectively generate mucosal and cellular immune responses [29,30]. For example, *Salmonella* Typhimurium strain X9558 delivers pneumococcal surface PspA protein to mucosal immune cells, thereby providing protection against pneumococcal challenge in mice [31,32].

*Salmonella* is a Gram-negative zoonotic bacteria that is a leading cause of food-borne diseases in developed countries and causes bloodstream infections in infants and elderly in developing countries [33]. *Salmonella* are frequently asymptomatic in livestock, but has been highly associated with mild and severe diarrhea in piglets [34,35]. In pigs, *Salmonella* Typhimurium is by far the most isolated serotype in the EU and US, followed by *Salmonella* Derby and *Salmonella* Arizona. It is useful for heterologous antigens or drug delivery vectors to protect intracellular pathogens, including viruses, due to their ability to induce both humoral and cellular immune responses in both mucosal and systemic compartments [36,37].

We previously developed an effective bacterial attenuation method using radiation mutation technology (RMT) that can easily reduce the virulence of pathogenic bacteria without genetic manipulation [38]. In the present study, we created a non-toxic *Salmonella* Typhimurium strain, named KST0666, from a clinical isolate (ST454 strain) using RMT and developed an effective FMDV VP1 protein stress-inducible expression system that delivers antigen to mucosal immune cells. Moreover, we evaluated the ability of this new antigen delivery vector system to elicit effective mucosal, humoral, and cellular immune responses.

## 2. Materials and Methods

### 2.1. Ethics Statement

The present study was performed in strict accordance with the recommendations in the guide for the care and use of Laboratory Animals of the National Institutes of Health. All animal experiments were ethically approved by the committee on the use and care of animals at the Korea Atomic Energy Research Institute (KAERI; approval No. IACUC-2018-007) and performed according to accepted veterinary standards set by the KAERI animal care center. To euthanize the mice, a CO_2_ inhalation method as specified by KAERI Institutional Animal Care and Use Committee guidelines was used.

### 2.2. Reagents

All chemical reagents used in the present study were purchased from Sigma-Aldrich (St. Louis, MO, USA).

### 2.3. Bacteria and Plasmid

Bacteria and plasmids used in the present study are listed in Table 1. *Salmonella* or *E. coli* were grown in Luria Broth (LB; Becton Dickson, Franklin Lakes, NJ, USA) in a rotary shaker at 200 rpm, 37 °C. The media were supplemented with kanamycin (50 μg/mL), chloramphenicol (15 μg/mL) or ampicillin (100 μg/mL) if required. The KST0666 strain was selected from *Salmonella* Typhimurium strain ST454 using RMT. Briefly, the wildtype (WT) strain was cultured in LB (OD = 0.5) and subjected to irradiation (1.2 kGy) using Co-60 gamma irradiator (point source AECL, IR-221, MDS Nordion International Co., Ltd., Ottawa, ON, Canada) for 1 h. Irradiated ST454 WT was spread on LB agar followed by incubation for 48 h at 37 °C. Then, 20 colonies were randomly selected for in vitro invasion assay. The isolate showing the lowest invasion ability was named KST0666.

VP1-expressing plasmid was constructed as described previously [38]. Briefly, codon optimized VP1 DNA of FMDV-type A/WH/CHA/09 (accession no. JF792355) was synthesized by Cosmo Genetech Inc. (Seoul, Republic of Korea) and cloned into pRECN plasmid. The resulting plasmid named pRECN-VP1 (KST0669) was transferred into KST0666 by electroporation (1.8 kV, 25 µF, and 200 Ω).

### 2.4. High-Throughput Sequencing Using an Illumina Platform

To investigate nucleotide substitutions, deletions, and insertions in the attenuated strain KST0666, its genome was sequenced using Illumina HiSeq 2000 (150 bp paired-end) with 825.98–fold coverage. The total length of read bases was 6,522,648,074 bp, which covered 98.09% length of the WT ST454 strain. The raw reads from the ST454 genome were mapped and aligned to the reference genome sequence using Burrows–Wheeler aligner (BWA-0.7.12) and Picard. Next, the genetic variants were detected using SAMTools (ver. 1.2).

### 2.5. Cell Invasion Assay

The porcine intestinal epithelial cell line IPEC-J2 was cultured in 48-well cell culture plates (SPL, Pocheon, Korea). When the cells reached about 90% confluence, the randomly selected irradiated *Salmonella* ST454 isolates (*n* = 20) were added to the cells at a multiplicity of infection (MOI) of 10 and incubated for 30 min at 37 °C. The cell monolayer was washed three times with phosphate-buffered saline (PBS) followed by cell lysis using 0.05% trypsin EDTA and 0.25% Triton X-100. Finally, the cell lysate was serially diluted with PBS, and the diluents were plated onto an LB agar plate to count the bacterial colonies. The invasion rate was calculated as [recovered colony-forming units (CFU)/original CFU] × 100%.

### 2.6. VLP_FMDV_ Purification

VLP_FMDV_ were purified using three SUMO fusion proteins in the same *E. coli* system containing pSMKVP0, pSMAVP1, and pSMCVP3 established at Lanzhou Veterinary Research Institute (LVRI; Lanzhou, China). The expression, purification, and proteolytic cleavage of fusion proteins were performed as described previously [14]. The assembled VLP_FMDV_ were identified using a Zetasizer-Nao instrument (DLS, Malvern Zetasizer-Nano ZS90; Worcestershire, UK) and transmission electron microscopy (Nikon, Tokyo, Japan).

### 2.7. Western Blot Analysis

Bacterial strains KST0666, KST0667, KST0668, and KST0669 were grown in LB and harvested at OD_600_ = 0.6–0.8, followed by separation of the supernatant and pellet. The pellets were lysed using 1% *v*/*v* Triton X100 and 0.1% *w*/*v* SDS in PBS, and the lysates were loaded and separated on 12% Bis-Tris BOLT gels (Invitrogen, San Diego, CA, USA), followed by transfer onto nitrocellulose membranes. The membranes were blocked with 5% skimmed milk in tris-buffered saline (TBS) for 60 min at room temperature. They were then incubated with rabbit anti-FMDV IgG (1:1000; LVRI) or rabbit polyclonal anti-DnaK IgG (1:3000; Abcam, Cambridge, UK) followed by incubation with an anti-rabbit IgG conjugated with horseradish peroxidase (HRP; 1:5000; Sigma-Aldrich) for 1 h at room temperature. The protein bands were visualized using Enhanced Chemiluminescent Western Blotting Substrate (Thermo Scientific, Waltham, MA, USA). Bio-Rad ChemiDoc™ Touch imaging system (Bio-Rad Laboratories, Hercules, CA, USA) and Bio-Rad CFX Manager software 3.1 (Bio-Rad Laboratories) were used for data acquisition and analysis.

### 2.8. Reverse Transcription-Quantitative PCR (RT-qPCR) Analysis

The mouse macrophage RAW264.7 cell line (Korea Cell Line Bank, Seoul, Republic of Korea) was cultured in six-well culture plates (SPL) with Dulbecco’s modified Eagle’s medium (DMEM; GIBCO, Carlsbad, CA, USA) without antibiotics and infected at an MOI of 10 with KST0666, KST0667, KST0668, or KST0669. After 2 and 18 h post-infection, the expression of VP1 was quantified by RT-PCR. The primers used are listed in Table 2. Total RNA isolation and cDNA synthesis was performed using an RNeasy^®^ mini kit (Qiagen, Hilden, Germany) and Primescript 1st strand cDNA synthesis kit (Takara Bio, Otsu, Japan), respectively. RT-PCR amplification and analysis were achieved using Bio-Rad CFX Connect^TM^ Real-Time System (Bio-Rad Laboratories) with SYBR Green Master Mix (Takara Bio). The relative gene expression was quantified using the 2^−ΔΔCt^ method [40]. The 16S rRNA *rrsH* was selected as a control to normalize the expression levels.

### 2.9. Fluorescence Microscopic Analysis

RAW264.7 cells were plated on imaging slides (µ-Slide 12-well, glass bottom, Ibidi GmbH, Munich, Germany), followed by infection with KST0669 at an MOI of 10. Unbound bacteria were washed out with PBS followed by fixation with 2% paraformaldehyde at 4 °C for 20 min and permeabilization with 0.1% Triton-X100 for 20 min. The cells were then washed three times with PBS and blocked with 3% bovine serum albumin (BSA; Sigma-Aldrich) in PBS for up to 2 h at room temperature. The cells were then incubated with rabbit anti-FMDV IgG, followed by staining with FITC-conjugated goat anti-rabbit IgG (Sigma-Aldrich). The nuclei were stained with 150 ng/mL 4′,6-diamino-2-phenylindole (DAPI; Thermo Scientific). The slides were washed with PBS and mounted with mounting medium (Dako, Carpinteria, CA, USA). All images were captured using an Olympus CX41 fluorescence microscope (Olympus Corporation, Tokyo, Japan).

### 2.10. Mice Experiments

All mice were obtained from OrientBio Inc. (Suwon, Republic of Korea). For comparison of 50% lethal dose (LD50), 6-week-old BALB/c mice (*n* = 4 per group) were injected intraperitoneally with different CFUs (ranging from 1 × 10^4^ to 3 × 10^8^ CFU/200 μL) of *Salmonella* strain ST454 WT or KST0666. The survival of infected mice was monitored for 14 days. To assess the ability of KST0669 to induce immune responses, 6-week-old BALB/c mice (*n* = 5 per group) were randomly assigned five mice per individually ventilated housing cages (Orient Bio, Sungnam, Korea) maintained in an animal BSL2 facility at 22–23 °C on a 12:12 light:dark cycle. Mice were administered orally with 100 µL of 10% sodium bicarbonate for 4 h prior to inoculate orally with either KST0668 or KST0669 (10^8^ CFU/100 µL) at 2-week intervals. Feces and blood samples were collected every week for analysis. Blood was obtained from the submaxillary sinus of the mice. Additionally, to verify the safety of KST0669 in mice, the spleen, liver, and mesenteric lymph node from KST0669-vaccinated mice were isolated on day 1, 2, 3, 5, and 7 post-inoculation, and the number of viable bacteria in each organ were determined by plating serially diluted homogenates or blood on LB agar plates. Bacterial counts were determined by enumeration of CFU. To further confirm the protective capacity of KST0669 vaccination, 6-week-old BALB/c mice (*n* = 5 per group) were orally vaccinated twice with KST0669 (10^8^ CFU) at 2-week intervals. On day 14, after the last vaccination, the mice were orally challenged with 10^6^ CFU of *Salmonella* Typhimurium LT2 and bacterial loads in the caecum, spleen, and blood were counted after serial dilution on LB agar plates on day 2, 4, and 6 post-infection. In the vaccinated group, mice mortality was observed and recorded for 2 weeks after the challenge.

### 2.11. Measurement of Mice Immunoglobulin

Feces and blood were collected from vaccinated mice (*n* = 5 per group) on day 0, 7, 14, 21, and 28 since the first vaccination as described above. Antibody titers in blood and feces were measured by enzyme-linked immunosorbent assay (ELISA). Briefly, 96-well immunoplates (SPL) were coated with 0.5 µg/mL of VLP_FMDV_, obtained from LVRI, in carbonate buffer (pH = 9.6) overnight at 4 °C. After blocking the plates with 2% BSA at room temperature for 45 min, they were washed with PBST (PBS containing 0.05% Tween-20) and then incubated with 100 µL of diluted mouse serum or fecal fluid at room temperature for 1 h. After washing the plates with PBST, HRP-conjugated goat anti-mice IgA, IgG, or IgM (1:3000; Sigma-Aldrich) were added to the wells and incubated for 30 min at room temperature. The plates were then washed with PBST, and 100 µL of 3,3′,5,5′-tetramethylbenzidine substrate reagent (BD Biosciences, San Diego, CA, USA) was added. After color development, 50 µL of 2N H_2_SO_4_ was added and the absorbance was measured at 450 nm using Bioteck Eponch Microplate Spectrophotometer (Biotek, Winooski, VT, USA).

### 2.12. Virus Neutralization Assays

FMDV neutralizing antibody titers of serum obtained from vaccinated mice were measured as described previously [41]. Briefly, isolated mouse serum was inactivated at 55 °C for 1 h and 50 µL serum was serially diluted in DMEM in a 96-well plate. Then, 50 µL of FMDV suspension (tissue culture infective dose = 100) was mixed with the serum and incubated for another 1 h at 37 °C. Subsequently, 50 µL of BHK-21 cell suspension (2 × 10^4^ cells) was added and incubated at 37 °C for 72 h and the cytopathic effects of each well were investigated. The virus neutralization titers of serum were calculated by the Spearman–Karber method [42].

### 2.13. Flow Cytometry

Then, 2 weeks after the last immunization, the spleen homogenates of immunized mice were filtrated through a cell strainer (40 µm; BD Biosciences) in RPMI 1640 medium (GIBCO) containing 10% fetal bovine serum (GIBCO) and then red blood cells (RBCs) were lysed with RBC lysis buffer (Sigma-Aldrich) for 3 min at room temperature. Single-cell suspension (2 × 10^6^ cells) was incubated with 10 μg/mL VLP_FMDV_, 2 μg/mL anti-CD28 monoclonal antibody (clone 37.51; eBioscience, San Diego, CA, USA), 0.5 μg/mL GolgiStop (eBioscience), and 0.5 μg/mL GolgiPlug (BD Biosciences) for 12 h at 37 °C. Next, the cells were washed with cold PBS and stained with live/dead staining kit (InvivoGen, San Diego, CA, USA), anti-CD4-BV450 (clone RM4-5; BD Biosciences), anti-CD8α-FITC antibodies (clone 53-6.7; BD Biosciences), anti-CD3e-APC-Cy7 (clone UCHT1; eBioscience) at 4 °C for 30 min. The cells were fixed using Cytofix/Cytoperm Plus Kit (BD Biosciences), washed, and then stained intracellularly with anti-mouse IFN-γ-PE (anti-mIFN-γ-PE; clone XMG1.2; BD Bioscience), anti-mIL-5-APC (clone TRFK5; BD Bioscience), and anti-mIL-17A-PE-Cy7 (clone eBio17B7; eBioscience) at 4 °C for 30 min. The stained cells were analyzed with MACSQuant VYB flow cytometer (Milteny Biotech, San Diego, CA, USA) and the results were analyzed with FlowJo software (TreeStar, Ashland, OR, USA).

### 2.14. Cytokine ELISA

For analysis of cytokine production, the culture supernatants of splenocytes prepared as described above were collected, and cytokine levels (IFN-γ, IL-5, and IL-17A) were measured with ELISA kit according to the manufacturer’s instruction (BD Bioscience).

### 2.15. Statistical Analysis

All data were presented as mean ± SEM of three independent experiments performed in triplicate. Results were analyzed by Student’s *t* test using the GraphPad Prism 6 software (San Diego, CA, USA). Comparisons among groups were also analyzed by two-way ANOVA followed by a post-hoc Tukey test. *p* values <0.05, <0.01, and <0.001 were considered statistically significant.

## 3. Results

### 3.1. Development of Attenuated Salmonella Strain Using RMT

**Figure 1:**
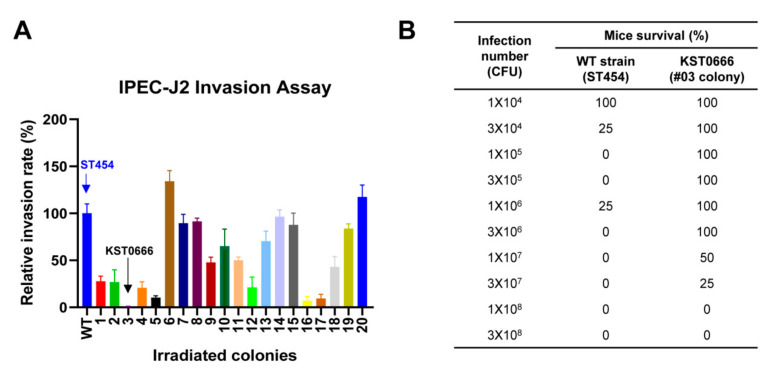
Selection of live attenuated *Salmonella* strain by radiation mutation technology (RMT). (**A**) The cell invasion ability of *Salmonella* Typhimurium ST454 WT and its irradiated mutants were tested in the porcine intestinal epithelial cell line IPEC-J2. IPEC-J2 cells were infected with ST454 WT or its irradiated mutants (*n* = 20) at a multiplicity of infection (MOI) of 10, bacterial viability in cell lysates that were serially diluted was counted at 30 min post-infection, and invasion rates were calculated as (recovered CFU/original CFU) × 100%. (**B**) The comparison of LD50 values measured in BALB/c mice (*n* = 4 per group) injected with either ST454 or KST0666 strain. The survival of infected mice was monitored for 14 days.

An attenuated *Salmonella* strain was developed using previously reported method [38]. After induction of mutations using γ-radiation in the WT strain (ST454), the isolates showing significantly reduced replication capacity in the porcine IPEC-J2 cells were selected as the attenuated candidate strains. Interestingly, most of the selected strains exhibited lower replication abilities than the parent strain (ST454), and among them, #03 colony showed the lowest replication rate compared to the other mutated isolates (Figure 1A). This lowest-replicating mutant strain without vector was selected for subsequent studies and named KST0666. In addition, the abbreviations of strains used in all in vitro and in vivo experiments were named as follows: attenuated *Salmonella* strain (KST0666) transformed with the vectors (pET28a or pRECN) lacking the insert was named as KST0667 and KST0668, respectively. KST0666 strain harboring pRECN-VP1 was named as KST0669.

Next, LD_50_ values were measured to determine whether KST0666 was sufficiently attenuated in comparison with WT strain (Figure 1B). The survival rates of mice intraperitoneally injected with WT or KST0666 (ranging from 1 × 10^4^ to 1 × 10^8^ CFU) and their survival rates were monitored. All mice infected with the WT strain died within 3 days, whereas the mice infected with lower than 1 × 10^7^ CFU of KST0666 exhibited 100% survival for the duration of the study (up to 14 days). Additionally, 50% and 25% of mice injected with 1 × 10^7^ and 3 × 10^7^ CFU of KST0666, respectively, survived, indicating that it was attenuated at least 500 times than its parent strain.

To analyze the location of mutations in KST0666, the complete genome of KST0666 was sequenced and compared to that of the WT strain as reference. As shown in Table 3, there were 16 genetic alterations, including 3 deletions, 1 insertion, and 12 point mutations detected in KST0666.

Among the 12 point mutations, 4 silent, 1 non-sense, and 7 missense mutations were found that may interfere with the functions of several damage repair or virulence genes relevant to the attenuation (Table 4).

Notably, two deletions and one insertion resulted in frameshift mutations through one nucleotide addition or loss. In total, two of them were detected in non-coding regions, and another was present in ST454-WT_04577 (Ribulose-phosphate-3-epimerase). In addition, an inframe deletion was detected in ST454-WT_00059 (putative protease SohB) wherein six nucleotides were missing. Taken together, these random alterations in nucleotides and amino acids might contribute to the attenuation of KST0666.

### 3.2. Expression of VP1 in Response to recN Promoter in KST0669

**Figure 2:**
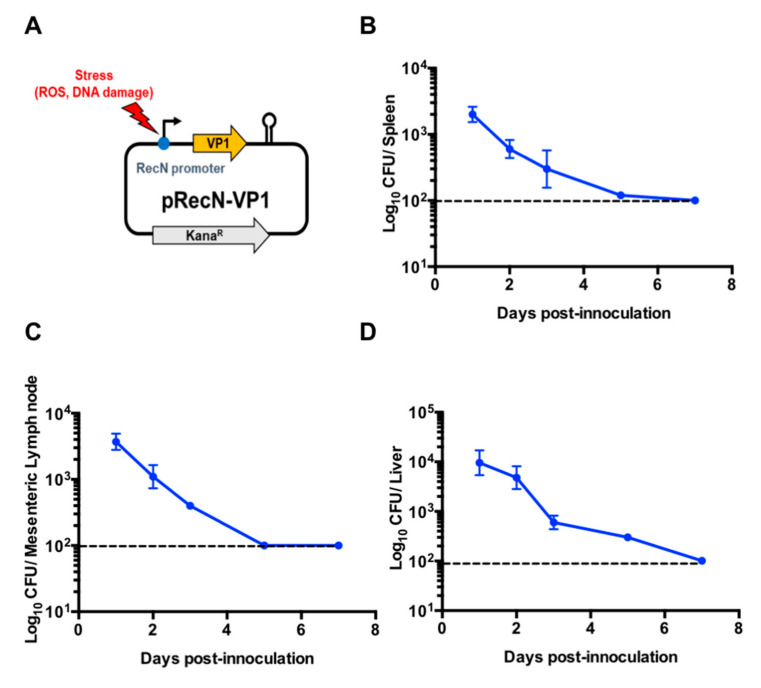
Virulence of *Salmonella* KST0666, containing pRECN-VP1 (KST0669). (**A**) Schematic plasmid map of stress-inducible VP1-expressing plasmid, pRECN-VP1. (**B**–**D**) In vivo virulence of KST0669. The colonization capacity of KST0669 in mice was measured in the spleen (**B**), the mesenteric lymph node (**C**), and the liver (**D**). BALB/c mice (*n* = 5 per group) were orally administrated 1 × 10^8^ CFU of KST0669, and euthanized on day 1, 2, 3, 5, and 7 post-infection. Bacterial viability was assessed in organ lysates that were serially diluted and plated on LB agar plates. Results are presented as mean ± SEM per group. The interrupted line indicates the undetectable level of bacteria. ROS, reactive oxygen species.

We previously developed a protein delivery system with a *recN* promoter under regulation of external stresses, such as DNA damage and reactive oxygen species [38]. Considering the immunogenicity of the VP1 capsid protein in FMDV infection, the VP1 protein-expressing system (pRECN-VP1) was constructed as shown in Figure 2A and transferred to KST0666 strain. To determine the safety of KST0666 harboring pRECN-VP1 (KST0669), BALB/c mice were orally inoculated with 1 × 10^8^ CFU of KST0669 and the numbers of colonized bacteria in organs were counted at day 1, 2, 3, 5, and 7 post-inoculation. There were no obvious clinical symptoms or body weight decline during the 7-day observation period post-inoculation. As shown in Figure 2, the invading *Salmonella* was completely eliminated from the spleen (Figure 2B), mesenteric lymph node (Figure 2C), and liver (Figure 2D) on day 7 post-infection, indicating the safety of this system in the mouse model.

**Figure 3:**
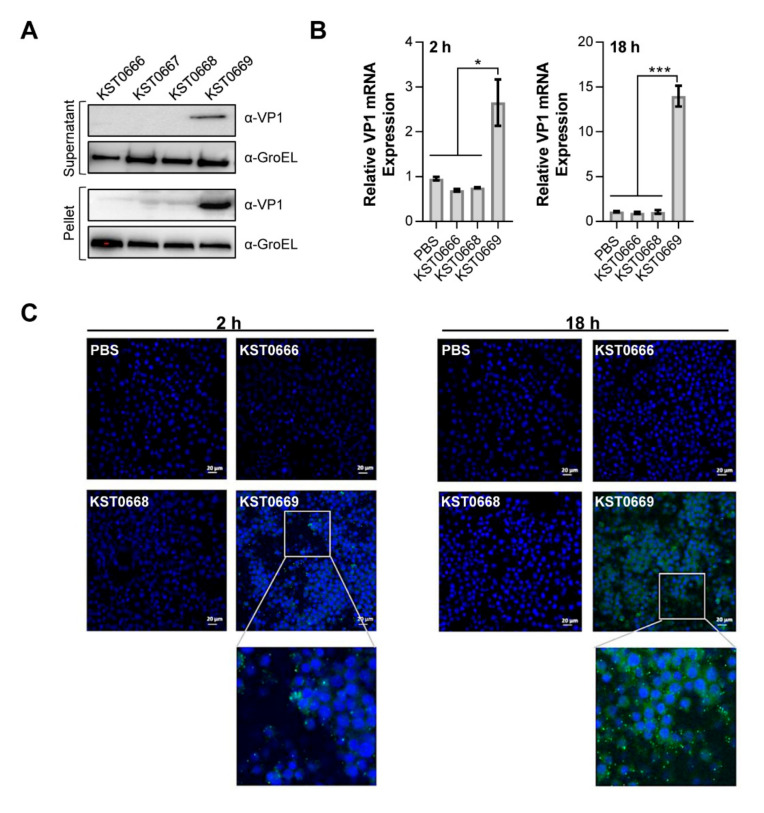
Expression of VP1 protein and mRNA in KST0669. (**A**) KST0666, KST0667, KST0668, or KST0669 were grown to mid-log phase and proteins were extracted from cell pellets and culture supernatants, after centrifugation. VP1 protein expression levels were measured with a rabbit anti-FMDV antibody and detected by western blotting. α-VP1: anti-VP1; α-GroEL: anti-GroEL. (**B**) VP1 mRNA expression levels in *Salmonella*-infected mouse macrophages. RAW264.7 cells (5 × 10^5^ per well) were infected with KST0666, KST0668, or KST0669 (MOI = 10) and phosphate-buffered saline (PBS)-treated RAW264.7 cells were used as negative control. Total mRNA was isolated at 2 and 18 h post-infection, and VP1-specific mRNA levels were measured using RT-qPCR. Data are expressed as mean ± SEM per group and analyzed by *t* test. *** *p* < 0.005, * *p* < 0.05. (**C**) Visualization of VP1 protein expression in *Salmonella*-infected RAW264.7 cells at 2 and 18 h post-infection. RAW264.7 cells (5 × 10^5^ per well) were infected with KST0666, KST0668, or KST0669 (MOI = 10). Cell nuclei were stained with DAPI (blue), and VP1 protein (green) was detected with rabbit anti-FMDV IgG and FITC-conjugated goat anti-rabbit IgG.

Subsequently, we examined the expression of VP1 in KST0669. As shown in Figure 3A, the VP1 protein was marginally expressed in bacteria under normal conditions; in contrast, under DNA damaging stress environment (irradiation with UV-B), an increased expression was detected both in the culture supernatant and cytoplasm of the lysed cell pellet.

We further quantified the expression of VP1 in macrophages, i.e., RAW264.7 cells infected with KST0669 (MOI of 10) using RT-qPCR. VP1 expression levels in the infected RAW264.7 cells were 2.65 ± 0.84- and 13.99 ± 2.01-times higher than in the PBS control group at 2 and 18 h post-infection, respectively (Figure 3B). To visualize VP1 expression in RAW264.7 cells, intracellular VP1 expressed by KST0669 was tagged with FITC-conjugated antibody and visualized by confocal microscopy (Figure 3C). At 2 h post-infection, a strong fluorescent signal was detected inside and outside the KST0669-infected RAW264.7 cells, and the intracellular expression of VP1 further increased at 18 h post-infection. These data suggested that KST0669 efficiently expressed the VP1 protein in antigen-presenting macrophages.

### 3.3. Mucosal and Humoral FMDV-Specific Immune Responses Elicited by KST0669 Vaccination

**Figure 4:**
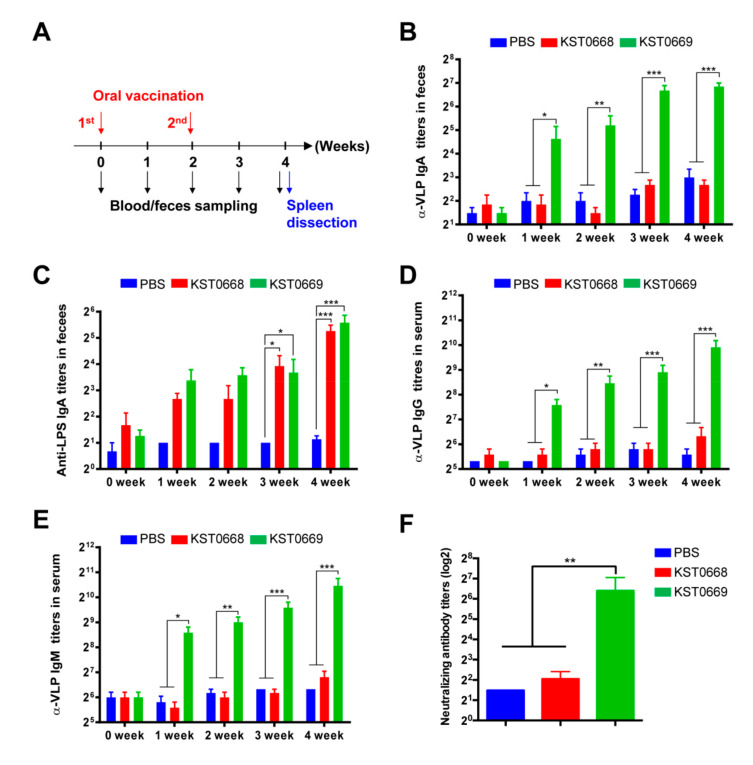
Humoral and mucosal immune responses induced by immunization with KST0669. (**A**) Schematic of the mouse experiment schedule. Mice (*n* = 5 per group) were orally administrated 1 × 10^8^ CFU KST0668 or KST0669 twice at 2-week intervals, and feces and sera were collected weekly for 4 weeks. (**B**,**C**) VLP_FMDV_-specific (**B**) or *Salmonella* LPS-specific (**C**) IgA in feces. VLP_FMDV_ (2 µg/mL) and *Salmonella* LPS (1 µg/mL) were immobilized on 96-well plate and IgA titer was measured by ELISA. (**D,E**) The serum levels of VLP_FMDV_-specific IgG (**D**) and IgM (**E**) were measured weekly. Data are expressed as mean ± SEM per group and analyzed by two-way ANOVA followed by a post-hoc Tukey test. (**F**) Neutralizing activity of FMDV in sera from PBS-, KST0668-, and KST0669-vaccinated mice. Pooled sera from immunized mice (*n* = 5 per group) at day 14 after the last vaccination were serially diluted and incubated with FMDV followed by incubation with BHK-21 cells. Cytopathic effects of the sera were measured 72 h after incubation. Data are expressed as mean ± SEM per group and analyzed by unpaired *t* test. *** *p* < 0.005, ** *p* < 0.01, and * *p* < 0.05. α-VLP: anti-VLP.

The primary site of FMDV invasion is the pharyngeal area, suggesting that mucosal immunogenicity plays a critical role in preventing FMDV replication and the damage caused by FMDV [43]. In order to evaluate if oral immunization of live KST0669 was able to elicit FMDV-specific mucosal and humoral immune responses, the fecal and sera samples of immunized mice were collected as shown in Figure 4A. In mice vaccinated with either KST0668 or KST0669, there was no difference in *Salmonella* lipopolysaccharide (LPS)-specific fecal IgA titers for 4 weeks indicating that the number of vaccinated *Salmonella* was similar in both groups (Figure 4C). However, VLP_FMDV_-specific fecal IgA, serum IgG, and serum IgM were not detected in the KST0668-vaccinated group, which was comparable with the PBS-injected control group (Figure 4B,D,E). On the contrary, the KST0669-vaccinated group showed significantly increased fecal IgA, serum IgG, and serum IgM titers compared to those in KST0668-vaccinated group in a time-dependent manner. The elevated antibody levels in the KST0669-vaccinated group indicated that our delivery system successfully delivered the VP1 antigen to immune cells and effectively induced both mucosal and humoral immunity.

Next, anti-FMDV neutralizing activity was examined to measure the functional activity of mouse sera from KST0699-vaccinated mice. As shown in Figure 4F, sera from PBS- or KST0668-vaccinated mice had marginal neutralizing antibody titers, whereas sera from KST0669-vaccinated mice had significantly higher levels of neutralizing antibody titers (87 ± 16.15) than that from KST0668-vaccinated mice. These results indicated that our delivery system effectively delivered VP1 to the immune cells, inducing mucosal, humoral, and functional FMDV-specific immune responses.

### 3.4. Higher FMDV-Specific T-Cell Immunity Induced by Oral KST0669 Vaccination

**Figure 5:**
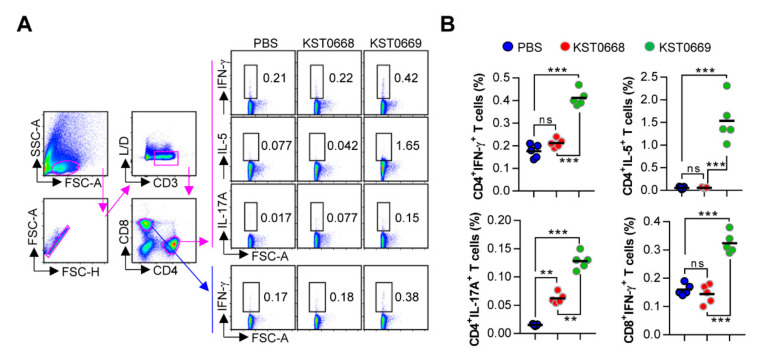
Analysis of VLP_FMDV_-specific CD4^+^ and CD8^+^ T-cell responses. Mice (*n* = 5 per group) were vaccinated orally twice with PBS, KST0668, or KST0669. (**A**,**B**) Spleen cell suspensions were re-stimulated with 10 μg/mL VLP_FMDV_ for 12 h and VLP_FMDV_-specific Th1 (IFN-γ-expressing CD4^+^ T cells), Th2 (IL-5-expressing CD4^+^ T cells), Th17 (IL-17A-expressing CD4^+^ T cells), and activated CD8^+^ T cells (IFN-γ-producing CD8^+^ T cells) were analyzed by intracellular cytokine staining based on the T cell-specific makers (anti-CD3, anti-CD4, and anti-CD8 antibodies). (**A**) The representative plot for Th1, Th2, Th17, and activated CD8^+^ T cells in spleen of PBS-, KST0668-, and KST0669-vaccinated mice. (**B**) The percentages of Th1, Th2, Th17, and activated CD8^+^ T cells in spleen of all vaccinated mice. The mean ± SD shown are representative of two independent experiments. *** *p* < 0.005, ** *p* < 0.01, ns = not significant.

As CD4^+^ and CD8^+^ T cells are important for protection against viral infections, including FMDV [24,25], we assessed the activation of cell-mediated immune response in KST0669-vaccinated mice. The single cell suspensions of splenocytes isolated from each orally vaccinated (twice at 2-week intervals) mice were re-stimulated with 10 µg VLP_FMDV_. Then, VLP_FMDV_-specific type 1 T helper (Th1; IFN-γ-expressing CD4^+^ T cells), Th2 (IL-5-expressing CD4^+^ T cells), Th17 (IL-17A-expressing CD4^+^ T cells), and activated CD8^+^ T cells (IFN-γ-expressing CD8^+^ T cells) were analyzed as described in Figure 5A. As shown in Figure 5B, significantly increased frequencies of IFN-γ^+^CD4^+^, IL-5^+^CD4^+^, and IL-17A^+^CD4^+^ cells were detected in KST0669-vaccinated group compared to those in PBS- and KST0668-vaccinated groups. Additionally, a significantly increased level of activated CD8^+^ T cells (IFN-γ^+^CD8^+^ T cells) in the KST0669-vaccinated group was found compared to that in the PBS- and KST0668-vaccinated groups.

**Figure 6:**
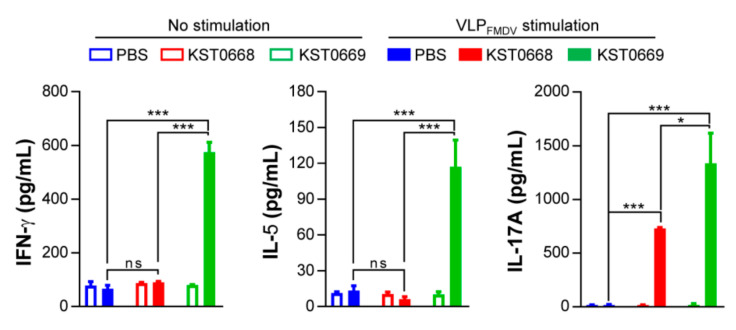
VLP_FMDV_-specific cytokine production in spleen cells of vaccinated mice. Mice (*n* = 5 per group) were vaccinated orally twice with PBS, KST0668, or KST0669. Single cell suspensions of splenocytes were treated with 10 μg/mL VLP_FMDV_ for 24 h, and supernatants were collected for determination of VLP_FMDV_-specific cytokines (IFN-γ, IL-5, and IL-17A) using ELISA. The mean ± SD shown are representative of two independent experiments. *** *p* < 0.005, * *p* < 0.05, ns = not significant.

VLP_FMDV_-specific cytokine levels in splenocytes isolated from each vaccinated group were measured by ELISA (Figure 6). Significantly increased levels of IFN-γ, IL-5, and IL-17A were detected in the KST0669-vaccinated group compared to those in PBS- or KST0668-vaccinated groups. This confirmed that our delivery system could induce FMDV-specific immune responses in Th1, Th2, and Th17 cells. Since Th17 cells and IL-17 cytokine from the mucosal compartment are reportedly associated with the transmission of bacterial and viral infection [44,45], a dramatic increase in FMDV-specific IL-17 secretion should be relevant in terms of providing protection against FMDV mucosal infections.

### 3.5. Protection Against Salmonella Typhimurium Infection Through KST0669 Vaccination

**Figure 7:**
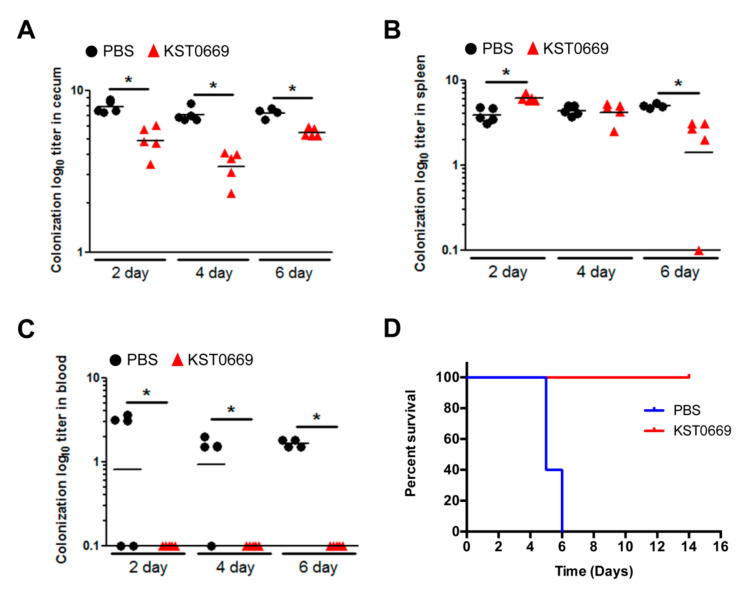
Protection against *Salmonella* infections. Mice (*n* = 5 per group) were orally immunized with PBS (black circle) or KST0669 (red triangle) twice at two-week intervals and then infected orally with 1 × 10^6^ CFU *Salmonella* Typhimurium LT2 strain. (**A**–**C**) Mice organs (cecum and spleen) and bloods were isolated at 2, 4, or 6 days post-infection. The CFU of bacteria in the cecum (**A**), the spleen (**B**), and blood (**C**) were counted. (**D**) Mice survival was monitored for 14 days. Data are expressed as mean ± SEM per group and analyzed by unpaired *t* test. * *p* < 0.05.

To confirm that KST0669 vaccination could protect against not only FMDV but also *Salmonella* infection, mice were immunized as shown in Figure 4A and infected orally with *Salmonella* Typhimurium LT2 (1 × 10^6^ CFU) for 2 weeks. The survival rates of mice were monitored for 2 weeks after infection. As shown in Figure 7D, all mice in the PBS-injected group died within 7 days after infection, whereas all mice vaccinated with KST0669 survived for 2 weeks. To assess the ratio of *Salmonella* invasion in mice, the numbers of *Salmonella* Typhimurium LT2 in the intestine, spleen, and blood was measured at 2, 4, and 6 days post-infection. As shown in Figure 7A,B, the KST0669-vaccinated group showed significantly lower viability of *Salmonella* Typhimurium LT2 colonization in the cecum and spleen than that in the PBS control group. In addition, *Salmonella* Typhimurium LT2 invasion in mouse blood increased day-by-day in the PBS group, whereas no bacteria were detected in the blood of the KST0669-vaccinated group (Figure 7C). These data indicate that the KST0669 vaccination effectively prevents lethal *Salmonella* Typhimurium challenge.

## 4. Discussion

Since FMD is one of the most economically devastating veterinary diseases, vaccination is the only effective strategy to prevent its disease burden and spread. Currently, chemically inactivated FMDV vaccine is widely used worldwide, but its protective efficacy has been reported as low as 60% [46]. Therefore, several next generation vaccines, such as mucosal, VLP, and peptide-based vaccines, have been developed as alternatives that may elicit superior immune responses against FMDV infection. Several studies have reported that the mucosal and T-cell dependent immune responses were induced by using live attenuated bacterial delivery systems, such as *Salmonella* Typhimurium, *Lactococcus lactis*, and *Bacillus subtilis* [47,48,49,50,51]. However, the present study is the first to report RMT as an effective and time-saving approach to develop live attenuated bacteria as an antigen delivery vector. Our novel *Salmonella* vector named KST0666 was highly attenuated both in vitro and in vivo, and could effectively transfer FMDV antigen (VP1 capsid) to the immune cells in mice. In addition, this new system can elicit a protective immune response against FMDV as well as *Salmonella* itself.

Radiation has been widely used to induce mutations in crop breeding [52,53]. In addition, several studies have reported the use of RMT in microorganisms to enhance the production of bioactive molecules in bacteria, yeast, or fungi [54,55,56]. For example, the production of bacterial cellulose, which has a variety of applications in medical and industrial field, was highly improved by radiation-induced mutagenesis in *Komagataeibacter hansenii* and *Gluconacetobacter xylinus* [57,58]. We previously developed attenuated *Salmonella* by applying RMT to improve its target activity against the tumor microenvironment [38]. Genome analysis of this *Salmonella* strain revealed that several mutations, including deletions and insertions, that are rarely found in chemical mutagenesis, were induced by RMT. Compared to the parent strain, KST0666, developed by RMT, was mutated at 16 sites with 3 deletions, 1 insertion, and 12 point mutations. Interestingly, a missense mutation was detected in the AdaA gene, which encodes a DNA repair enzyme that plays a role in repairing the adaptive response to alkylation damage [59]. Another missense mutation was found in the LptG gene that is involved in the transport of LPS from inner membrane to the cell surface [60]. The combination of several identified candidate mutant genes can trigger attenuation of KST0666, but the exact mechanism of attenuation remains still unclear. It is also necessary to investigate the mechanism of attenuation due to single or multiple gene mutations through genetic manipulation.

To enhance the cellular immune response of FMDV vaccines, many approaches have been developed. The viral vectors can easily express and deliver immunogenic structural proteins of FMDV to induce diverse immune responses in vector-infected cells [61]. Previously, a human replication-defective adenovirus subunit vaccine (Ad5-O1Man) was genetically modified to express the capsid and capsid-processing proteins of FMDV, and consequently induced neutralizing antibodies in swine [62]. Recently, another Ad5 vaccine co-expressing the VP1 capsid gene with the 3C protease gene of FMDV A12/119/Kent/UK/32 was shown to protect direct contact homologous FMDV transmission in cattle [63]. Live attenuated bacterial strains as a vaccine vector have been widely studied for their advantages in stimulating mucosal and cellular immunity [26]. *Lactococcus* is generally considered safe and is known to be able to induce adjuvant effects non-specifically through macrophage activation [64]. For example, *Lactobacillus plantarum* with the pSIP411-VP1 plasmid induces VP1-specific IgG and mucosal secretory IgA, providing protective immunity against FMDV infection in guinea pigs [65]. Our *Salmonella* system was shown to be similar, in terms of eliciting an immune response, to previously reported bacterial vector systems. However, an additional advantage of our system is that the KST0669 vaccine can elicit an immune response against both infecting organisms FMDV and *Salmonella*. This is significant as *Salmonella* is a commonly recognized zoonotic pathogen and may colonize the gut of pigs or cattle, contaminating the carcasses during the slaughter process [66].

Furthermore, this is the first study to use VLPs for evaluating FMDV vaccine efficacy. FMDV-specific humoral and cellular immune responses are conventionally measured using live FMDV or killed FMDV prepared in a laboratory with BSL3 facility. There was no need for a BSL3 facility in the present study because it was possible to evaluate the efficacy of the vaccine using VLPs prepared in *E. coli*. In addition, this method was more quantitative then using live FMDV, so it was possible to ensure accuracy in vaccine evaluation.

## 5. Conclusions

The present study demonstrated a novel strategy to develop a live attenuated bacterial vector system using RMT. This system effectively delivered the viral antigen to immune cells in vivo to elicit both FMDV- and *Salmonella*-specific immune responses. In addition, this is the first study to successfully evaluate the VLP_FMDV_-specific humoral and cellular immune responses against FMDV. Collectively, our data provide important information for developing vector-based FMDV vaccines and evaluating the efficacy of a novel concept of FMDV vaccines in a (BSL1) laboratory.

## Figures and Tables

**Table 1:** Plasmids and strains used in this study.

Types	Name	Characteristics	Origin
Plasmids	pET28a(+)	Expression vector, Kan^R^	Novagene
	pRECN	derived from pET28a(+), with strong expression of promoter *recN* from *E. coli*	[38]
	pRECN-VP1	derived from pRECN with VP1 gene	in this study
Strains	KST0666	attenuated *Salmonella*	in this study
	KST0667	KST0666 harboring pET28a(+)	in this study
	KST0668	KST0666 harboring pRECN	in this study
	KST0669	KST0666 harboring pRECN-VP1	in this study
	LT2	*Salmonella* Typhimurium	[39]
	ST454	*Salmonella* Typhimurium clinical strain isolated from pigs	in this study

**Table 2:** Primers used in this study.

Primers	Sequence (5′–3′)
recN-F	AAC CAT GGT TAA TAT CCG CAA TAC AC
recN-R	TTG AAT TCT GTG CAT TCC TCT CCC
VP1 amplification-F	AAG AAT TCA CTA CTT CGA CGG GGG AAA GCG
VP1 amplification-R	GGC GGT TAA ACA GAG CTT AAC AAG TGA GCG GCC GCA A
VP1 diagnosis-F	CCG GTT ACT GCG ACA CTA GT
VP1 diagnosis-R	TTT AAC CGG CGC CAC AAT TT
VP1-RT-PCR-F1	GAT CCG GTT ACT GCG ACT GT
VP1-RT-PCR-R1	ACA ATA GGT TTC CGC CCG TT

**Table 3:** Summary of the mutational spectrum observed in KST0666.

Mutation Type	Deletion	Insertion	Point	Total
Silent	0	0	4	4
Missense	0	0	7	7
Nonsense	0	0	1	1
Frameshift	2	1	0	3
Non-frameshift	1	0	0	1
Total	3	1	12	16

**Table 4:** Mutated genes in KST0666.

Protein ID	Position	Homologous Protein	Mutant Type
ST454_00059	55692	Putative protease SohB	inframe deletion
ST454-00105	108541	Respiratory nitrate reductase 1 alpha chain	synonymous variant
ST454_00266	285135	Chemotaxis protein CheA	synonymous variant
ST454-00371	395359	Cobalt-precorrin-7 C(5)-methyltransferase	synonymous variant
ST454-00871	888709	Anaerobic dimethyl sulfoxide reductase chain B	missense variant
Non-coding region	1471061		frameshift variant
ST454-02089	2175005	Hypothetical protein	missense variant
ST454-02736	2873993	Bifunctional transcriptional activator/DNA repair enzyme AdaA	missense variant
ST454-02754	2892634	Hypothetical protein	missense variant
ST454-02790	2937098	Lipopolysaccharide export system permease protein LptG	missense variant
ST454-03046	3209927	Secretion monitor	missense variant
ST454-03672	3848803	Putative lipoprotein ChiQ	synonymous variant
Non-coding region	4023820		frameshift variant
ST454-04101	4326390	O-acetyl-ADP-ribose deacetylase	nonsense
ST454-04336	4590924	Hypothetical protein	missense variant
ST454-04577	4819484	Ribulose-phosphate 3- epimerase	frameshift variant

## Data Availability

The data that support the findings of this study are available on request from the corresponding author.
